# Territoriality of Giant Otter Groups in an Area with Seasonal Flooding

**DOI:** 10.1371/journal.pone.0126073

**Published:** 2015-05-08

**Authors:** Caroline Leuchtenberger, William E. Magnusson, Guilherme Mourão

**Affiliations:** 1 Graduate Program in Ecology, Instituto Nacional de Pesquisas da Amazônia—INPA, Manaus, Brasil; 2 Laboratório de Vida Selvagem, Embrapa Pantanal, Corumbá, Brasil; University of Florida, UNITED STATES

## Abstract

Territoriality carries costs and benefits, which are commonly affected by the spatial and temporal abundance and predictability of food, and by intruder pressure. Giant otters (*Pteronura brasiliensis*) live in groups that defend territories along river channels during the dry season using chemical signals, loud vocalizations and agonistic encounters. However, little is known about the territoriality of giant otters during the rainy season, when groups leave their dry season territories and follow fish dispersing into flooded areas. The objective of this study was to analyze long-term territoriality of giant otter groups in a seasonal environment. The linear extensions of the territories of 10 giant otter groups were determined based on locations of active dens, latrines and scent marks in each season. Some groups overlapped the limits of neighboring territories. The total territory extent of giant otters was correlated with group size in both seasons. The extent of exclusive territories of giant otter groups was negatively related to the number of adults present in adjacent groups. Territory fidelity ranged from 0 to 100% between seasons. Some groups maintained their territory for long periods, which demanded constant effort in marking and re-establishing their territories during the wet season. These results indicate that the defense capacity of groups had an important role in the maintenance of giant otter territories across seasons, which may also affect the reproductive success of alpha pairs.

## Introduction

Most social species defend territories within their home range in an attempt to have exclusive access to important resources [1– 3]. However, territories may be more or less exclusive and some overlap with neighboring intruders may occur [4– 7]. An important strategy to avoid conspecific rivals is to inform ownership to intruders, by such means as chemical signaling, which is an efficient communication tool, even in the absence of the signaler, (e.g. [8–10]). Scent-marking may inform intruders about the composition and size of the group, and the aversion for marks left by strangers may constrain owners to their territories [9]. Species adopt different patterns of territory marking and, in heterogeneous habitat, signals are commonly concentrated in areas where the threat of intrusion is highest [9–13].

Territoriality carries costs and benefits, which are commonly affected by the abundance and predictability of food in time and space [14]. The resource-dispersion hypothesis (RDH) suggests that territory sizes of carnivores are determined by resource dispersion, while group size is related to the quality of patches [15]. Assuming that territory size and shape represent an economic optimum [14], the minimum defensible territory would contain enough resources to maintain a minimum breeding unit, and areas with more abundant resources will support additional individuals [15–17]. Therefore, when resources are widely distributed, the size of territories may be related to the total metabolic needs of the group [18] and consequently to group size [7, 15, 19], although the relationship between group and territory size is potentially complicated by other factors that benefit group-living, such as hunting and breeding cooperation [20].

In seasonal environments, some species maintain territories only during periods in which important resources are available [5]. As groups select and establish territories in a given area, the order of territory establishment may affect the final size of the territory and the fitness of the owners for the entire territorial season, since the first groups that establish territories during this period will have territories of optimal size, and later groups will have to establish their territories in the remaining space [5, 21]. Therefore, neighbor pressure may prevent expansion of territories and result in conflicts and territory overlap [4–6, 13, 22–24]. In such situations, the size of exclusive territories may have a negative correlation with intruder pressure [4], with greater overlap in areas with more conflicts. The pressure of neighboring groups may have negative consequences because of agonistic encounters [9, 10, 25], which may affect the maintenance of territories [26]. Groups that remain in the same territory for longer periods will become more familiar with the area, which may also improve fitness and defense capacity, since owners learn to explore their territory more efficiently and to defend areas that are more frequently invaded by intruders [27].

Giant otters live in cohesive groups ranging from two to 20 individuals, which cooperate in the care of the offspring of the dominant pair [28]. Giant otter groups mark their territories with scent-marks and communal latrines, which are located at dens and campsites along the banks of water bodies [28, 29]. Agonistic encounters result in fighting and loud vocalizations when a group or a solitary individual is detected within the territory of a resident group [29– 32]. Estimates of territory sizes of giant otter groups have been made during dry seasons in Guyana and Suriname, and in the Amazon and Paraguay River basins [28, 33–38]. However, little is known about the territoriality of giant otters during the rainy season, when groups are believed to leave their territories and follow fish dispersing into flooded areas [28, 39, 40]. We analyzed long-term territoriality of giant otter groups in a seasonal environment with the aim of quantifying the effects of season on territorial defense behavior, territory exclusivity and fidelity, and territory size. Since territoriality implies costs associated with defense and intruder pressure, we expected that giant otter groups would deposit more chemical signals at the borders of their territories and that larger territories would have more chemical signals. Since defense capacity may increase with group size, we hypothesized that larger groups would maintain larger territories, and that the size of territories would be limited by the number of intruders present in adjacent groups. As territoriality is benefited by resource concentration and abundance, groups are expected to increase their territory size and decrease the investment in chemical signaling during the flooding season, when resources are dispersed over larger areas.

## Material and Methods

### Ethics Statement

Since the giant otter is an endangered species, all field activities, data collection and procedures for capturing and marking the animals in the study area were authorized under license no. 12794/4 issued by ICMBio, the Federal Environmental Agency of Brazil. The study was approved by the Ethics Committee at the National Institute of Amazonian Research under the number 028/2013. All methods were performed following the guidelines of the American Society of Mammalogists for the use of wild mammals in research [41] and the recommendation listed in the license. The same team, including two veterinarians with experience with the species, performed all the capture and surgical procedures. We also avoided to capture and approach of groups with cubs or pregnant females. Giant otter groups are already used to the presence of boats, since the area is visited by tourists and fishermen. During observations, we followed the groups at a distance of 10–100m, depending on the perceived shyness and general reaction of the group to observer presence, to avoid any unnecessary disturbance.

### Study area

The Pantanal is a large wetland covering approximately 160 000 km^2^, located in areas of western Brazil, Bolivia and Paraguay. The hydrological regime is regulated by seasonal rains, which fall mostly between November and March and result in the inundation of almost 80% of the area [42].

### Data collection

From June 2009 to June 2011, we studied home-range size and habitat selection of ten giant otter groups inhabiting stretches of the Miranda and Vermelho Rivers in the southern Pantanal. In this study we use the linear home range estimates based on the extreme locations of each group that have been published previously [37, 40], to examine aspects of the long-term stability of territories of giant otter groups in a seasonal environment. The lengths of stretches of river used by each group were based on 43 to 965 locations over 6 to 24 months of monitoring.

Ten giant otter groups were monitored (G1-G4, G8-G13) for 8–10 consecutive days every month, interspaced by two to three weeks, along 119 km of the Vermelho (19°34'S, 57°01'W) and Miranda Rivers (19°36'S, 57°00'W) in the southern Pantanal. Monitoring was undertaken by boat or on land, during the daytime (05:00–19:00), when we searched for individuals or groups, active dens, campsites, scent-marks and other signs (see [43]), and registered the locations with a global positioning system receptor (Garmin Etrex, Inc., Olathe, KS). Giant otters were video recorded (Canon HF-200), which allowed identification of their natural individualistic marks on the throat, and their sex and behavior whenever possible. The hierarchical status of individuals within the group was identified according to their behavior and other cues. Alpha males were prominent in the defense of the group and territory demarcation and alpha females were lactating during the reproductive season and were more involved with the care of cubs and with the coordination of group activities. Other individuals were classified as subordinates.

Two dominant males and one subordinate male from different giant otter groups (G2, G10 and G12) were captured, chemically immobilized and implanted intraperitoneally with radio-transmitters (M1245B, Advanced Telemetry System, Isanti, Minnesota, weigh 42g), following [44]. Radiotagged giant otters were released after they recovered from the anesthesia at the place of capture or near their group. For more details about the capture, surgical procedures and animal care of this study see the methods section in [40]. Radio-tracking was conducted from November 2009 to June 2011, varying from eight to 12 months of monitoring for each marked group (G2—from Nov/09 to Jun/10; G10 and G12—from Jul/10 to Jun/11), totaling 153 days of telemetry monitoring. Telemetry signals were monitored with a Yagi antenna (RA-17, Telonics, Mesa, Arizona) attached to a 2.5-m pole and connected to a TR4-receiver (Telonics). We undertook two aerial surveys with a fixed-wing aircraft (CESSNA-182) to locate groups during the flooding season.

We measured the level of the Miranda River (variation from 126 to 481 cm) daily at a fixed station (19°34°S, 57°01°W), which allowed us to recognized two dry seasons (June-December 2009 and July 2010-January 2011) and two wet seasons (January-June 2010 and February-June 2011) during the study period.

### Data analysis

We measured the linear extent of territories of all groups observed, based on the extreme locations of active dens, latrines and scent-marks in each season. As the defended linear territories coincided with the linear home-ranges for some groups, the linear territories reported here (Table 1) largely coincided with the linear home-ranges for those groups provided for Leuchtenberger et al. ([40]; Table 2). We also measured the exclusive stretches within the territories, which were defined as the core area defended only by the resident group that did not overlap with areas defended by adjacent groups [1]. We calculated territory overlap between groups as the proportion of the territory of one group that overlapped the territory of another group. These overlaps are asymmetrical between groups [45].

**Table 1:** Percentage of territories (in rows) overlapped by the neighboring group’s territory (in columns) during each season (dry season of 2009, wet season of 2010, dry season of 2010, wet season of 2011) from June 2009 to June 2011, in the southern Pantanal, Brazil.

	Groups	G1	G2	G3	G4	G8	G9	G10	G11	G12	G13
	G1 (8)	100	38.93	0	0	10.22	0				
Dry	G2 (3)	69.23	100	0	0	0	0				
season	G3 (5)	0	0	100	31.69	0	18.78				
2009	G4 (7)	0	0	26.16	100	0	0				
	G8 (8)	15.04	0	0	0	100	39.09				
	G9 (6)	0	0	17.54	0	36.38	100				
	G1 (8)	100	27.35	0	0		0	24.11			
Wet	G2 (3)	34.09	100	0	0		0	0			
season	G3 (3)	0	0	100	*		0	0			
2010	G4 (7)	0	0	*	100		0	0			
	G9 (3)	0	0	0	0		100	0			
	G10 (9)	*	0	0	0		0	100			
	G1 (5)	100	0	0	0			0	0	0	
	G2 (2)	0	100	0	0			0	0	0	
Dry	G3 (9)	0	0	100	*			16.79	0	0	
season	G4 (7)	0	0	*	100			0	0	0	
2010	G10 (15)	0	0	10.36	0			100	0	0	
	G11 (4)	0	0	0	0			0	100	*	
	G12 (3)	0	0	0	0			0	*	100	
	G1 (4)	100		0	0		0	0	0	0	0
	G3 (9)	0		100	*		0	*	0	0	0
	G4 (7)	0		*	100		0	0	0	0	0
Wet	G9 (2)	0		0	0		100	48.31	0	0	*
season	G10 (11)	*		6.86	0		19.21	100	0	0	0
2011	G11 (4)	0		0	0		0	0	100	*	*
	G12 (3)	0		0	0		0	0	18.26	100	0
	G13 (3)	0		0	0		*	0	*	0	100

Group size is indicated in parentheses beside the group ID. The territories of groups with <20 locations were not included in the analyses and are indicated by asteisks.

**Table 2:** Territory total extent (TE, km) and exclusive territory (ET, km) of ten giant otter groups monitored by radio-telemetry (RT) and direct observations (DO), during four seasons (dry season of 2009, wet season of 2010, dry season of 2010, wet season of 2011), from June 2009 to June 2011, in the southern Pantanal, Brazil.

		Dry 2009		Wet 2010		Dry 2010		Wet 2011	
	Groups	TE	ET	TE	ET	TE	ET	TE	ET
	G2	10.5	3.0	21.4	13.1	0.7*	0.7*		
RT	G10			7*	0*	22.2**	19.9**	23.9	16.1
	G12					1.1	1.1	8.1	6.6
	G1	17.8¹	9.1	23.1** ¹	15.8	20.1* ¹	20.1*	4.2*	4.2*
	G3	14.2¹	7.3	14.8¹	14.8	13.71	11.4	4.9* ¹	4.9* ¹
	G4	17.2¹	12.7	6.7* ¹	6.7	0.3* ¹	0.32*	4.1* ¹	4.1* ¹
DO	G8	12.1¹	5.5						
	G9	13¹	6.0	8.5* ¹	8.5			9.5	6.9
	G11					9.4* ¹	9.4*	1.8* ¹	1.8*
	G13							1.6* ¹	1.6*
	Median	13.6¹	6.7	18.1	13.9	7.4	6.3	9.5	6.9

¹Data from [40].

*estimates should be considered with caution, as they are based on few locations (<20).

** there was an expansion and/or shift of territory, which may have lead to overestimated territory sizes, and these values were not used to calculate medians.

We combined latrines and isolated scent-marks as chemical-signal sites. The density of chemical signals was estimated for each group along the linear extent of river within the exclusive and overlapping areas and these estimates were averaged for dry and wet seasons. We used a two-way-ANOVA to test the difference between the densities of chemical-signals sites between exclusive and overlap areas and seasons.

Group size was considered the number of individuals in the group during each season, including juveniles 6-months-old or more, since they were usually already integrated in the daily activities of the group. Potential intruder pressure was estimated by summing the number of adults and sub-adults (>1 year old) present in adjacent groups during each season. We estimated averages of the total-territory extent, the exclusive-territory extent, group size, intruder pressure and the number of chemical-signal sites for each group during each season (dry and wet seasons). We also included in these calculations the estimates of territory size of giant-otter groups monitored during 2006 and 2007 by [37] in the same area. We used ANCOVA models to estimate (1) the effects of territory size and season on the number of chemical-signal sites, (2) the effects of group size and season on territory size, and (3) the effect of number of adult otters in neighboring groups and season on exclusive-territory size of groups. In all cases, we first checked the assumption of slope parallelism of ANCOVA by testing a preliminary model including the interaction term between the explanatory variables [46]. If the interaction term was not significant (*P*>0.05), we excluded it and ran the ANCOVA without the interaction. If the interaction term was significant, we tested the simple effect of the continuous explanatory variable separately for each season.

Agonistic events were video recorded during the study period. However, agonistic events occurred only at the limits of territories when other groups were present. Therefore, we could not predict when an agonistic event would take place and observations were incidental. We classified these events as "fights" when individuals of different groups fought or chased each other, and "warning vocalizations", when a group emitted agonistic vocalizations (e.g. screams and hahs, see [47]) to another group or when invading the borders of neighboring territories. Since we did not observe enough agonistic events to make confident conclusions of seasonal patterns, we present this data only for purposes of discussion.

We used the percentage overlap of total extent of the territory of a group during one season with the total territory extent from the previous season as an index of territory fidelity. Territory fidelity was estimated only for groups that were monitored in more than three months during each season. As giant otters in this area had been monitored since 2006 by our team, we used the available data [37, 40] to evaluate long-term changes in group territories. There was no significant relationship between estimated territory size and number of observations of the groups during the dry (*P* = 0.102) or wet seasons (*P* = 0.285). However, due to the small sample size (n<20 per season), territory estimates from some groups were excluded from analyses involving seasonality.

## Results

The ten monitored groups had a mean of six individuals per group (2–15 individuals), totaling 77 individuals (20 females, 26 males and 31 indeterminate) inhabiting the study area, which resulted in a linear density of 0.42 individuals/km. Almost all groups overlapped their territory borders with neighboring groups. Overlap ranged from 0 to 69% of linear extensions during both dry and wet seasons (Table 1).

The linear density of chemical-signal sites did not differ significantly between exclusive and overlapped territories (F_(1,27)_ = 0.431, *P* = 0.517) or seasons (F_(1,27)_ = 0.802, *P* = 0.379). There was no interaction between territory size and season (*P* = 0.899) when predicting the number of chemical signal sites. The number of chemical-signal sites increased linearly with territory size (number of chemical-signal sites = 1.55 + 0.858 ∙ territory size -3.295 ∙ seasons; t_partial_ = 4.884, *P* <0.001), but did not differ between seasons (t_partial_ = -1.401, *P* = 0.185).

**Fig 1:**
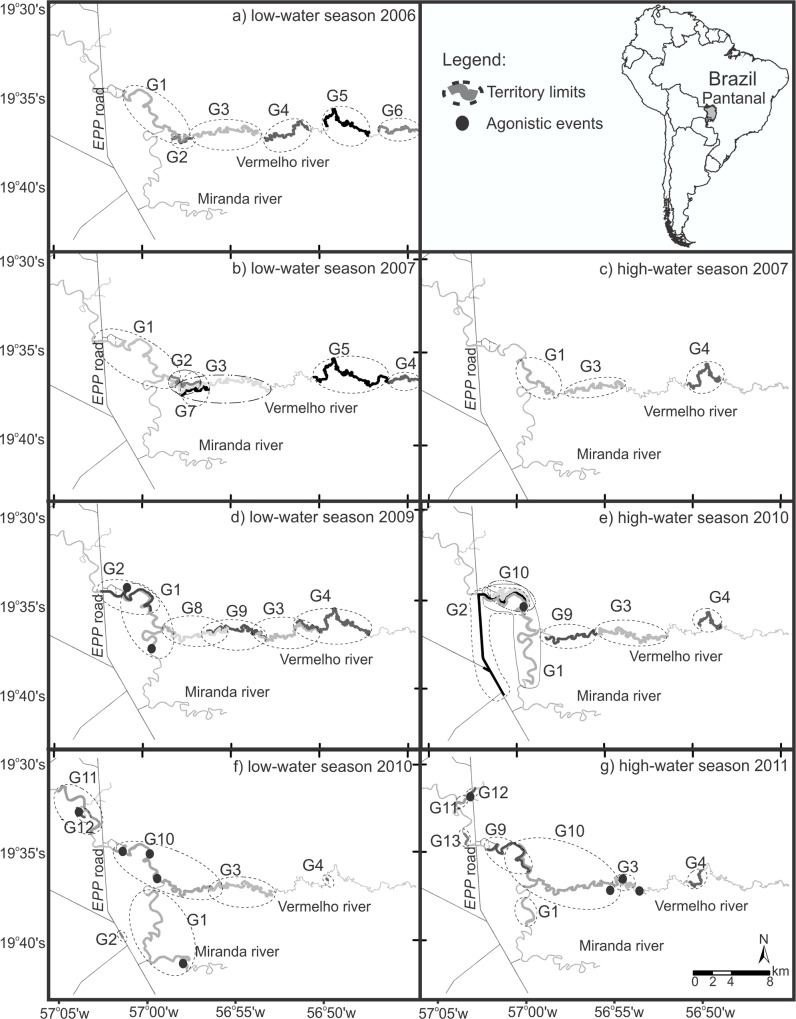
Territory extent of 13 giant otter groups (G1-G13) monitored between July 2006 and November 2007 [37], and from June 2009 to June 2011 [this study], on the Miranda and Vermelho Rivers, in the southern Brazilian Pantanal. Parts a, b and c were modified from [37]. Linear territories are represented by the gray lines along the rivers. The ellipses do not represent actual areas used, but are used to better vizualize boundaries and overlaps.

During the dry seasons, linear territories ranged from 1.1 to 17.8 km (n = 7 groups, Table 2), excluding the estimates for two groups, which had few locations and one new group that was establishing its territory within the season. The wet-season linear territories ranged from 8.1 to 23.9 km (n = 5 groups), as some groups extended their former ranges into the flooded areas or artificial lakes beside stretches of the Estrada Parque Pantanal (EPP) road (Fig 1). The extent of the exclusive territories varied from 1.1 to 12.7 km during the dry seasons and from 6.6 to 16.1 km during the wet seasons (Table 2). There was no interaction (*P* = 0.669) between group size and season, as explanatory variables of territory size. ANCOVA (F_(2, 13)_ = 11.67, *P* = 0.001, R² = 0.64) indicated that territory size increased linearly with group size (territory size = 1.434 + group size + 6.599 ∙ season; t_partial_ = 4.356, *P*<0.001) and was larger during wet season (t_partial_ = 2.910, *P* = 0.012). The effects of the number of adult and sub-adult otters in neighboring groups interacted with the season (t = 2.827, *P* = 0.015) as explanatory variables of exclusive territory size. The exclusive territory size was linearly and negatively affected by the number of adult neighboring otters during the dry seasons (territory size = -1.732 + neighboring otters; F_(1, 8)_ = 8.487, *P* = 0.019, r² = 0.52), but not during the wet seasons (F_(1,4)_ = 0.509, *P* = 0.515).

**Fig 2:**
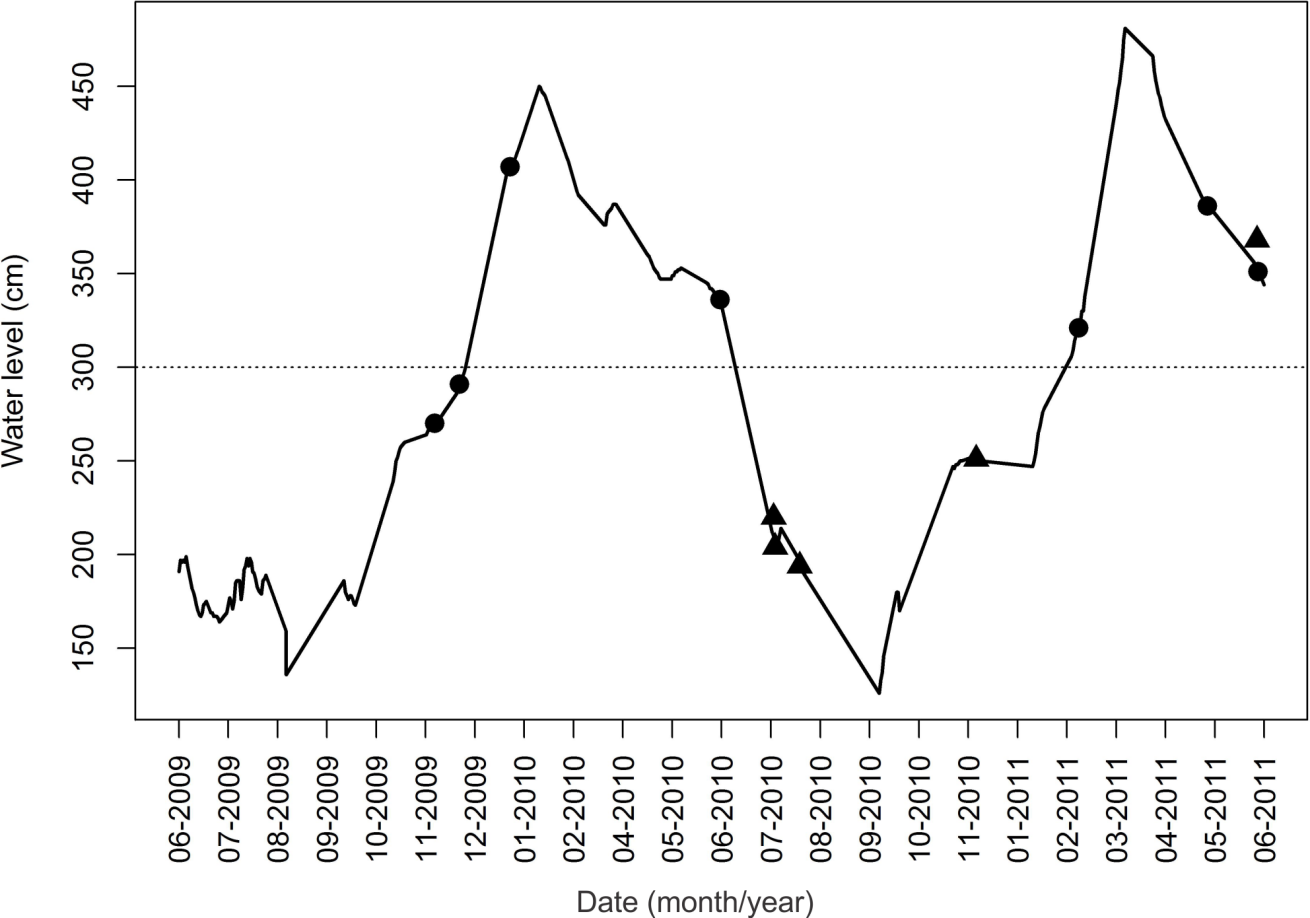
Water level in the Miranda River in the Brazilian Pantanal during the study. The dotted line indicates the limit between dry (river within its banks) and wet (floodplain inundation) seasons. Agonistic events between giant otters groups are indicated by triangles (fights) and circles (warning vocalizations).

Although most overlapped areas were not used simultaneously by more than one group, we witnessed 12 agonistic events between groups, which seemed to occur more often at the boundaries (Fig 1), including warning vocalizations (n = 7) and fights (n = 5). Most of the fights we saw occurred during dry periods, while the warning vocalizations were more frequent during floods, but the number of observations is too small to allow generalizations (Fig 2).

Territory fidelity varied from 0 to 100% between seasons (Table 3). Three groups (G2, G9 and G12) changed their territories completely between seasons (Fig 1). Group G8 was observed in the area only during the dry season of 2009. Group G10 expanded its territory from the wet season to the dry season of 2010 pushing group G1 up the Miranda River. Nevertheless, group G1 maintained 15% of its territory between the dry seasons of 2009 and 2010. Groups G1 and G3 have been observed in the study area since 2002 [36] and group G4 established its territory in 2006 [37], with changes in territory location thereafter (Fig 1). Group G2 was first observed in 2006, but the dominant male was substituted four times in the following two years [37]. During the wet season of 2010, we sighted group G2 in the same site that the group used during the dry season of 2008.

**Table 3:** Territory fidelity (% overlap of territories among seasons) of six giant otter groups between consecutive seasons (columns 1–3) and between same season (wet or dry) in different years (columns 4–5), monitored from June 2009 to June 2011, in the southern Pantanal, Brazil.

Groups	DS2009-WS2010	WS2010-DS2010	DS2010-WS2011	DS2009-DS2010	WS2010-WS2011
G1	26.30	35.53		15.09	
G2	43.2	100		0	
G3	51.72	73.78	100	27.41	
G9					0
G10			71.09		
G12			0		

Dry season = DS; wet season = WS.

## Discussion

The linear density of 0.42 ind/km of river observed for the giant otters in the study area has been stable since the surveys conducted in 2002 in the same area [36], reinforcing the suggestion that the species in this area has achieved carrying capacity [32, 37]. This density is within the range reported for Guyana, Suriname and the Amazon Basin, which varied from values as high as the 1–2 ind/km [28, 38] to about 0.2 ind/km [33, 48]. Otters may increase the intensity of territorial-defense behaviors when at high densities [22, 33], and giant otter groups may defend the entire extent of their home ranges, even though some boundaries overlap [28].

In our study, some giant otter groups overlapped the boundaries of neighboring territories and both groups alternately scent-marked these areas, sometimes on the same day, which could have confounded the real limits of group territories. Nevertheless, territory boundaries of giant otter groups seem not to be static, it would be useful to investigate territory dynamics using territorial-interaction models, with short time windows [49]. Demarcation in overlapped territories has been considered as attempts of animals to expand their territories [5, 7, 16]. Some social species increase their investment in the defense of borders to maximize the chance of being detected by intruders [9–11, 13]. In our study, the total territory extent and the extent of exclusive territories were larger during the wet seasons than during the dry seasons. Furthermore, the number of giant otter chemical-signal sites was positively correlated with territory length in both seasons and the density of chemical-signal sites did not differ between overlapped and exclusive territories ([37], this study), indicating that groups spend proportionally more time and energy to mark their territory as it increases in size. Although scent-marking is energetically expensive, giant-otter groups commonly forage throughout their entire territory every day, and may expend almost 10% of their daily active time in marking their territory [50]. For species such as *Lutra lutra*, inhabiting areas where flood-pulses occur within hours, scent-marks may signal priority of use of resources for other members of the group [3]. However, for a species as cohesive as the giant otter, inhabiting areas with seasonal flood-pulses, the distribution of chemical signals throughout the territory may be related to defense and reduction of intrusion, since a sparsely marked territory could be considered a vacant area by neighboring groups [9].

Territory size of giant otters was correlated with group size in both seasons, despite the increase in territory size during the wet season, which may suggest that larger groups increase the size of their territories more during flooding, when resources are more dispersed. Although this relationship is not common for social carnivores living in heterogeneous habitat [7, 15, 16, 51], this could be related to the need of larger groups to access more resources [15, 19], as there is a relationship between metabolic needs and home range size for most carnivores [2, 18]. The addition of individuals to the group may also improve defense capacity [9, 16, 24, 52], favoring the acquisition of larger territories.

During the dry season, the extent of exclusive territories of giant otter groups was negatively related to the number of adults present in adjacent groups. The pressure of intruders may restrict the expansion of territories, and consequently exert a negative effect on exclusive-territory size [4–6, 24]. However, there was no relationship between intruder pressure and exclusive-territory size during the wet season, probably because resources become widely distributed during floods, attenuating the pressure of neighboring groups with the decrease in density of otters along water bodies.

Fights between giant otter groups are highly vocal and may lead to severe injuries or death of individuals and the disintegration of the group [30, 31, 53]. In our study, agonistic events (fights and warning vocalizations) were common at the borders of territories and areas of territory overlap, and appeared to be more frequent during the dry season, while warning vocalizations were more frequent during floods. Smaller groups seemed to avoid fights in overlap areas. We have witnessed cases of large groups invading the territory of smaller groups, which remained hidden in marginal swamps or ponds until the invaders left the area. However, hiding is more practicable at wet season when there are many ways to avoid detection or to escape, which may account for the lower rate of fights between groups during the wet season. Agonistic encounters, as well as scent-marking, may constrain each group to its territory [9, 25]. As scent marking may inform intruders about the composition and identity of the group [9, 54, 55], the behavior of mixing feces and urine in communal latrines could be a strategy to hide the information about the size of the group, in order to make it more difficult for larger groups to identify weakly protected territories.

Territory fidelity ranged from 0 to 100% between seasons. Site familiarity may be a strategy to promote continuous access to key resources and enhance the owners´ fitness [27]. Also, in seasonal environments, such as the Pantanal, the establishment of territories before important resources become available appears to help a group maintain its territory in the following seasons [5, 21]. However, in these seasonal areas, the maintenance of territories could be difficult, as flooding can submerge marks and border limits, and allow access to new areas not yet settled. Despite these difficulties, in our study area, some groups maintained their territory for long periods (> 7 years), which demanded effort in marking and re-establishing their territories during the wet season.

Defense capacity apparently had an important role in territory maintenance of giant otter groups across seasons, and negative experiences during fights may lead a group to abandon its territory. During the wet season of 2010, a larger group (G10) overlapped and ultimately took over the territory from group G1, which was forced to dislocate up river and settle a new area. Other groups apparently were forced to leave their high-quality territories and settle new ones in sub-optimal or marginal areas ([32, 40], this study). The shift of one group from the river to a marginal habitat (group G2) seems to have caused it to reduce in size, with the death of the cubs and the dispersal of the only subordinate. The reproductive success of giant otter alpha pairs maybe affected by their capacity to maintain a territory in a high-quality environment for a long time, and by increasing the number of helpers in the group. Giant otter groups commonly increase in size through the philopatry of offspring [28]. However groups with unrelated members were observed in the study area [37, 56], which may be an efficient strategy to improve the success of a giant otter group and individual fitness, as survival of unrelated members would be enhanced by the strength of the group while they were gathering experience and body condition before trying to establish their own group.

## Supporting Information

S1 License Creative Commons Attribution license for reproduction of figure. Permission from the Editor of the Sociobiology Journal to publish an adaptation of Fig 2 published in the paper "Social Organization and Territoriality of Giant Otters (Carnivora: Mustelidae) in a Seasonally Flooded Savanna in Brazil. Sociobiology. 2008; 52(2): 257–270” under the Creative Commons Attribution 3.0 license.
(PDF)

S1 Related Manuscript Leuchtenberger C, Oliveira-Santos LGR, Magnusson W, Mourão G. Space use by giant otter groups in the Brazilian Pantanal. J Mammal. 2013; 94(2): 320–330.
(PDF)
