# Supplementary material for: Territoriality of Giant Otter Groups in an Area with Seasonal Flooding
Source: PLoS One. 2015 May 8;10(5):e0126073. doi: 10.1371/journal.pone.0126073 (PMC4425482; doi:10.1371/journal.pone.0126073)
Supplement: S1 License — Permission from the Editor of the Sociobiology Journal to publish an adaptation of Fig 2 published in the paper "Social Organization and Territoriality of Giant Otters (Carnivora: Mustelidae) in a Seasonally Flooded Savanna in Brazil. Sociobiology. 2008; 52(2): 257–270” under the Creative Commons Attribution 3.0 license. (PDF) [file pone.0126073.s001.pdf]

---------- Forwarded message ----------
From: Evandro do Nascimento Silva <evandro@uefs.br>
Date: 2015-02-22 20:38 GMT-03:00
Subject: Re: license for publishing
To: Caroline Leuchtenberger <caroleucht@gmail.com>

Dear Caroline Leuchtenberger,

In reply to your previous message, the Journal Sociobiology, ISSN 0361-6525 hereby states its agreement to the publication in the journal PLOS One of Figue 2 published in the paper "Social Organization and Territoriality of Giant Otters (Carnivora: Mustelidae) in a Seasonally Flooded Savanna in Brazil" in 2008 in the Sociobiology, under the Creative Commons Attribution License (CCAL) CC BY 3.0

With best wishes,

--
Dr. Evandro do Nascimento Silva
Universidade Estadual de Feira de Santana
Associate Editor - Sociobiology ISSN 0361-6525
Av. Transnordestina s/n   Novo Horizonte
Feira de Santana - BA   CEP 44036-900
