# Supplementary material for: Territoriality of Giant Otter Groups in an Area with Seasonal Flooding
Source: PLoS One. 2015 May 8;10(5):e0126073. doi: 10.1371/journal.pone.0126073 (PMC4425482; doi:10.1371/journal.pone.0126073)
Supplement: S1 Related Manuscript — Space use by giant otter groups in the Brazilian Pantanal. J Mammal. 2013; 94(2): 320–330. (PDF) [file pone.0126073.s002.pdf]

Journal of Mammalogy, 94(2):320–330, 2013

Space use by giant otter groups in the Brazilian Pantanal

CAROLINE LEUCHTENBERGER,* LUIZ GUSTAVO RODRIGUES OLIVEIRA-SANTOS, WILLIAM MAGNUSSON, AND GUILHERME MOURÃO

Laboratory of Wildlife, Embrapa Pantanal, Rua 21 de Setembro, 1880, CEP 79320-900, Corumbá, MS, Brazil (CL and GM)
Department of Ecology, Universidade Federal do Rio de Janeiro, Av. Carlos Chagas Filho, 373–Sala A027, CEP 68020–Ilha do Fundão, CEP 21941-902, Rio de Janeiro, RJ, Brazil (LGROS)
Graduate Program in Ecology, Instituto Nacional de Pesquisas da Amazônia (INPA), Av. André Araujo, 2936, CEP 69083-000, Manaus, AM, Brazil (CL and WM)

* *Correspondent: caroleucht@gmail.com*

Giant otters (*Pteronura brasiliensis*) live in groups that seem to abandon their territories during the flooding season. We studied the spatial ecology of giant otter groups during dry and wet seasons in the Vermelho and Miranda rivers in the Brazilian Pantanal. We monitored visually or by radiotelemetry 10 giant otter groups monthly from June 2009 to June 2011.We estimated home-range size for all groups with the following methods: linear river length, considering the extreme locations of each group, and fixed kernel. For the radiotracked groups, we also used the *k*-LoCoh method. Spatial fidelity and habitat selection of giant otter groups were analyzed seasonally. On the basis of *k*-LoCoh (98%) method, home-range sizes during the wet season (3.6–7.9 km^2) were 4 to 59 times larger than during the dry season (0.1–2.3 km^2). Home-range fidelity between seasons varied among giant otter groups from 0% to 87%, and 2 radiotagged groups shifted to flooded areas during the wet seasons. Giant otter groups were selective in relation to the composition of the landscape available during the dry seasons, when the river was used more intensively than other landscape features. However, they seemed to be less selective in positioning activity ranges during the wet season. During this season, giant otters were frequently observed fishing in the areas adjacent to the river, such as flooded forest, grassland, and swamps.

Key words: habitat selection, home range, landscape selection, *Pteronura brasiliensis*, site fidelity

© 2013 American Society of Mammalogists
DOI: 10.1644/12-MAMM-A-210.1

Animals adopt different strategies to deal with spatial and temporal heterogeneity of environmental features. Most species constrain their activities to an area on the landscape defined as a home range, which comprises areas used in diverse ways for survival, reproduction, and other activities that maximize fitness (Krebs and Davies 1997; Powell 2000). Some core areas are used more intensely within the boundaries of the home range and commonly contain refuges and more defendable food sources (Kernohan et al. 2001; Samuel et al. 1985). The maintenance of the home range in space and time is favored by a cognitive map (Spencer 2012) that provides site familiarity, which enhances the owners' fitness, increasing their ability to forage and to move rapidly and safely in the area (Stamps 1995).

Some landscape features are used more by a species than their proportional availability in the environment (Aebischer et al. 1993; Johnson 1980). However, under highly seasonal fluctuations, changes in habitat and resource availability may induce a shift in the animal's spatial organization and habitat use through different seasons (Arthur et al. 1996; Humphrey and Zinn 1982). Availability and abundance of food resources, together with the metabolic needs of each species, seem to be the most important variables determining the home range size and habitat selection of carnivores (e.g., Dillon and Kelly 2008; Macdonald 1983; Valenzuela and Ceballos 2000). Space use by semiaquatic mammals is strongly affected by the availability of water bodies and prey, and such relationships have been reported for several species of otters (Blundell et al. 2000; Garcia de Leaniz et al. 2006; Kruuk 2006; Melquist and Hornocker 1983). In places with well-defined hydrological cycles, flooding increases the amount of water in the landscape and may result in the dispersal of fish assemblages across vast

www.mammalogy.org

flooded areas (Wantzen et al. 2002; Winemiller and Jepsen 1998), which may attract fish predators and induce predictable movement patterns of the piscivores.

Giant otters (*Pteronura brasiliensis*) feed mainly on fish, and information on their spatial ecology is limited to direct observations during the dry season, when groups maintain linear territories along water bodies (Duplaix 1980; Evangelista and Rosas 2011a; Laidler 1984; Leuchtenberger and Mourão 2008; Ribas 2004; Schweizer 1992; Tomas et al. 2000; Utreras et al. 2005). Groups build dens and campsites with communal latrines throughout their home ranges that are used for resting, scent-marking, and rearing cubs (Duplaix 1980; Leuchtenberger and Mourão 2009; Lima et al. 2012). During the rainy season, giant otters seem to relinquish their territories to follow spawning fish into the flooded forest and swamps, and to search for emergent sites for building dens and campsites (Duplaix 1980). Seasonal shifts in movement patterns can increase home-range sizes of giant otter groups, which have been estimated to be 4 to 13 times larger during the rainy season (Utreras et al. 2005). However, in the absence of fluctuating water levels, giant otter groups seem to maintain their territories throughout the year (Laidler 1984).

The Pantanal is an extensive wetland located near the center of South America and it is subject to a strong annual flood pulse, which is considered to be the most important ecological phenomenon for the maintenance of local biodiversity (Alho 2008). Giant otters are locally abundant and distributed throughout this region (Leuchtenberger and Mourão 2008; Tomas et al. 2000). In this paper, we examine home-range size, home-range fidelity, and habitat selection of giant otters in the Brazilian Pantanal on the basis of direct observations and radiotelemetry, with the aim of answering the following questions: Is home-range size during the wet season larger than in the dry season? Do giant otter groups show home-range fidelity within and between seasons? Do habitat-selection patterns differ between seasons?

Material and Methods

From June 2009 to June 2011, we monitored 10 giant otter groups in the Vermelho River (19°34′S; 57°01′W) and a stretch of the Miranda River (19°36′S, 57°00′W), totaling 119 linear km of river, in the southern Pantanal of Brazil. The annual precipitation in the region is about 1,200 mm, with most of the rain falling between November and March (Hamilton et al. 1996). Due to the low declivity and seasonal inundation, almost 80% of the plain undergoes transition from terrestrial to aquatic habitat during the rainy season (Alho 2008). We measured the level of the Miranda River every day at a fixed station (19°34′S, 57°01′W), and it varied from 126 to 481 cm during the study period. Flooding tended to be abrupt, and the transition from wet to dry occurred within a few weeks. On the basis of the river-level measurements, we recognized 2 dry seasons (June–December 2009 and July 2010–January 2011) and 2 wet seasons (January–June 2010 and February–June 2011) during the study.

We monitored giant otter groups by boat, using a video camera (Canon HF-200, Lake Success, New York) to record individual natural marks on the throat of otters and their behaviors. This allowed us to identify the sex, position in the group hierarchy, group composition, and other details about the individuals. The location of individuals, groups, dens, latrines, and other vestiges were registered by a global positioning system (GPS) receptor (Garmin Etrex, Inc., Olathe, Kansas).

Between November 2009 and July 2010, we undertook three 10-day field trips to capture and implant radiotransmitters in individuals from different groups of giant otters. In each campaign, we first searched for active dens suitable for setting traps (i.e., dens with 1 or few entrances relatively free of entanglements of roots and vegetation). We blocked the den entrance with a funnel-shaped net late at night, as described by Silveira et al. (2011), and waited in the vicinity of the den to capture the individuals in the early morning. We captured 2 dominant males (from groups G2 and G12) and 1 adult subordinate male (group G10). The mean weight of captured individuals was 30.97 kg ($SD = 1.75$) and the mean total body length 178 cm ($SD = 6.25$).

We chemically immobilized the animals after capture using a dosage of 2.0 mg/kg of a combination of tiletamine and zolazepam (Zoletil, Virbac, Carros-Cedex, France) and applied a complementary dosage of 1.5 mg/kg ketamine hydrochloride 10% (Vetaset, Fort Dodge, Campinas, Brazil) combined with 0.25 mg/kg midazolam (Dormonid, Roche, Jaguaré, Brazil). Radios were implanted intraperitoneally by a registered veterinarian. During surgery, we applied 0.5 ml of intramuscular penicillin (Pentabiótico Veterinário, Fort Dodge Animal Health, Campinas, Brazil) and a subcutaneous dosage of 2 mg/kg of anti-inflammatory/analgesic (Ketoprofen 1%, Merial Animal Health, Paulínea, Brazil). We examined each captured individual for general body condition, photographed their throat markings, and took body measurements. The radiotransmitter (M1245B, Advanced Telemetry System, Isanti, Minnesota) weighed 42 g (~0.1% of body weight). All handling and surgical procedures followed the guidelines of the American Society of Mammalogists for the use of wild mammals in research (Sikes et al. 2011), and were authorized under license No. 12794/4 of the Brazilian Institute of Environment and Renewable Natural Resources. We released the radiotagged giant otters, after they recovered from the anesthesia, at the place of capture or near their group.

We radiotracked animals by boat or walking on the bank with a Yagi antenna (RA-17, Telonics, Mesa, Arizona) attached to a 2.5-m pole and connected to a TR4-receiver (Telonics). One group (G2) was monitored from November 2009 to June 2010, and 2 groups (G10 and G12) from July 2010 to June 2011, totaling 153 days of monitoring. Radiotagged animals were monitored from 0500 h, when almost all members of the group had left the den, to 1900 h or when the whole group had entered the den, during 8–10 consecutive days every month. On 2 occasions, when the tagged animals were not found for 2 consecutive months, we undertook aerial surveys with a fixed-wing aircraft (CESSNA-182) to locate

them. Once located from the ground, we followed the animals as silently as possible, keeping a distance that apparently did not disturb their behavior. We recorded locations with the GPS every 30 min when the group could be seen until we lost the radio signal. Since groups G10 and G12 had territories near each other, we monitored these groups in alternate periods (0500–1200 h or 1230–1900 h). We undertook nocturnal monitoring irregularly, but these data were not considered for home-range and habitat-use analyses, as movements were very limited at night. We used only locations recorded more than 10 days after capture in the analyses to avoid abnormal behavior due to the effects of capture and handling.

Home range.—Removal of sequential data to increase independence of locations can reduce the biological meaning of the information (Blundell et al. 2001; De Solla et al. 1999; Reynolds and Laundre 1990; Rooney et al. 1998). Also, giant otters cover much of their home range every day, so observations taken over 6–13 h per day tend not to be clustered in a limited part of the home range. Therefore, we considered all sequential locations (n = 2,321) acquired by radiotelemetry for home-range analysis, as well as some visual locations of the group G2 (n = 38) made before the capture event.

To allow comparison with other studies, we estimated home range for all groups as linear river length (RL) within the extreme locations of each group, which is commonly used to estimate giant otter linear home range (Evangelista and Rosas 2011a), and the fixed-kernel estimator with ad hoc estimation of the h value. For the radiotracked groups, for which we obtained more locations, we also used the k-LoCoh method (Getz and Wilmers 2004). Because groups sometimes shifted their areas from one season to another (Leuchtenberger and Mourão 2008), we stratified the home-range estimates by seasons in cases where we had more than 20 locations for a given group in a given season. All home-range analyses were undertaken in the R software (R Development Core Team 2011), using the packages ade4 (Thioulouse et al. 1997), adehabitat and adehabitatHR (Callenge 2006), gpclib, maptools (Lewin-Koh et al. 2009), rgdal (Keitt et al. 2010), rgeos (Renard and Bez 2005), and shapefiles (Stabler 2003). We calculated 98% and 95% isopleths for k-LoCoh analyses and 95% isopleths for the kernel estimator of total home-range size and the 50% isopleths to delimit core areas.

We measured the linear extension of river (RL) and/or other water bodies, such as ponds, streams, or flooded areas along roads, within the extreme locations of each monitored group using the GPS TrackMaker software (Ferreira 2004). For the kernel analysis, we tried to use the least-square cross-validation method, but this analysis did not converge. Therefore, we chose the h-value of $h = 80$ and $h = 100$, respectively, for the dry and wet seasons analyses of all groups, as they resulted in kernel-contour shapes that visually better accommodated the group locations.

To evaluate if we had enough locations to determine the home-range areas of radiotracked giant otter groups, we plotted the cumulative estimated LoCoh 100% areas chronologically. For this analysis, we fixed the number of nearest-neighbor locations (k) to 5. However, to estimate the appropriate k for calculation of the group's home-range area, we followed the procedure described in Ryan et al. (2006). That is, we plotted the home-range areas on the basis of 100% of locations, calculated with k values varying from 2 to 30 (100% isopleths) for each group. The asymptote of the 3 radiotagged groups was estimated to be approximated at $k = 16$, which was the value used for the k-LoCoh analyses.

We overlapped the home ranges and core areas (estimated with the kernel and LoCoh methods) of groups that were monitored in consecutive seasons to estimate the percentage of area fidelity. These were estimated with ArcMap 10.0 software (ESRI 2010), using the clip function. We also calculated the daily speed of each radiotracked group by season, dividing the daily mean of the Euclidean distance traveled among consecutive locations by the respective mean of time interval.

Selection of landscape features.—Here we use habitat to mean a category of physical environment that occurs in a circumscribed area that is available to an organism or group of organisms. In this sense, habitats include areas that may never be used by the organism under study. Used in this way, habitats are not necessarily related to particular organisms, and do not exist as inherent natural objects in the landscape, but are merely convenient categories that humans use to get a preliminary understanding of the spatial relationships of organisms to their environment. We created 3 landscape-category maps, representing 3 seasons (dry, wet 2010, and wet 2011), due to the differences in the flood levels of the wet seasons during the study period. We classified Landsat TM5 (NASA Landsat Program 2009, 2010, 2011) satellite images within seasons using the Kmedia method in the Spring v.4.3.3 software (Câmara et al. 1996). We digitalized an image taken during the dry season of 2009 in Google Earth and classified it in ArcMap 10.0 software (ESRI 2010). The wet-season images were overlapped on this dry-season image, recovering some landscape-unit types that could not be classified automatically. We used 6 landscape-unit categories: river; pond (comprising permanent and temporary freshwater ponds, and artificial ponds created during the construction of roads or water reservoirs used for cattle); swamp (water bodies that act as a transition between the aquatic and terrestrial, normally found at the edge of ponds, streams, and rivers, and that are dominated by grasses sometimes including isolated trees and shrubs); seasonally flooded grassland (seasonally flooded plains, including the grasslands); forest (riparian forest, semideciduous forest, and/or woodland savanna); and grassland (nonflooded matrix of grasses and herbs, we also included in this class roads and a few riparian human communities that were established in areas that originally had this vegetation cover).

We analyzed landscape-category selection of giant otter groups using a log-ratio compositional analysis (Aebisher et al. 1993) with 2,000 permutations in the R 2.13 software, using the packages adehabitat (Callenge 2006), maptools (Lewin-Koh et al. 2009), raster (Hijmans and van Etten 2010), rgdal

Table 1.—Home-range size (km^2) and overlap area (km^2 and %) between dry (DS) and wet (WS) seasons of 10 giant otter groups (ID = G1–G4, G8–G13; G size = range of number of individuals that composed the group during the monitoring period) monitored by radiotelemetry (RT) and direct observations (DO) from June 2009 to June 2011 in southern Pantanal, Brazil. Home ranges were estimated with *k*-LoCoh (isopleths 98%, 95%, and 50%) and kernel ad hoc ($h = 80$ for dry season and $h = 100$ for wet season) methods.

						k-LoCoh									Kernel					
					DS			WS			Overlap			DS		WS		Overlap	
	ID	G size	Period	Days	Locations	98%	95%	50%	98%	95%	50%	98%	95%	50%	95%	50%	95%	50%	95%	50%
RT	G2	3	14 August 2009–10 June 2010	79	965	1.0	0.8	0.03	3.9	3.1	0.003	0.8 (78%)	0.6 (73%)	0.005 (17%)	2.7	0.3	5.3	0.06	1.4 (51%)	0
	G10	9–15	4 August 2009–21 June 2011	81	793	2.3	1.7	0.03	7.9	7.9	0.5	2 (87%)	1.4 (85%)	0.004 (13%)	2.4	0.3	12.0	0.06	2.0 (83%)	0.007 (2%)
	G12	2–3	4 August 2009–20 June 2011	69	591	0.1	0.04	0.004	3.6	2.3	0.01	0	0	0	0.5	0.1	4.3	0.3	0.03 (6%)	0
	G1	5–8	5 June 2009–21 June 2011	57	177										3.2	0.3	3.9	0.2	2.8 (87%)	0
	G3	3–9	3 June 2009–17 March 2011	26	71										3.2	0.1	1.0	0.2	0.5 (17%)	0.01 (8%)
	G4	7	3 June 2009–17 June 2011	11	43										1.1	0.1	-	-	-	-
DO	G8	8	2 June 2009–15 December 2009	22	80										2.3	0.3	-	-	-	-
	G9	2–6	15 August 2009–18 June 2011	31	72										2.1	0.2	-	-	-	-
	G11	4–6	22 July 2010–18 May 2011	9	18										-	-	-	-	-	-
	G13	3	15 February 2011–11 May 2011	6	7										-	-	-	-	-	-

(Keitt et al. 2010), rgeos (Renard and Bez 2005), and shapefiles (Stabler 2003). We undertook landscape-category selection analysis for 8 groups (G1–G4, G8–G12) within the 2nd and 3rd levels proposed by Johnson (1980), which are the home-range area selected by each group in the study area, and space use (locations) of the groups within their home ranges. A buffer of 2 km was incorporated around each location of giant otters during the monitoring period in the study area (Blundell et al. 2001) using ArcMap 10.0 software. This buffer range was considered the study area for compositional analysis within home ranges. For home-range availability, we used the fixed-kernel contours with ad hoc estimation of the h values. We undertook eigen analysis of selection ratio as described by Callenge and Dufour (2006), which assigns scores to each giant otter group and habitat, resulting in a measure of habitat selection for each group. We counted the number of dens and campsites built by giant otter groups in each landscape feature to analyze the proportion of refuge and site locations in each habitat.

Results

From June 2009 to June 2011 ($n = 188$ days), we visually monitored 10 giant otter groups ($n = 361$ locations), totaling 77 individuals in groups (20 females, 26 males, and 31 indeterminate) with an average of 6 individuals per group (varying from 2 to 15; Table 1). Three giant otter males (2 dominant and 1 subordinate) of different groups (G2, G12, and G10) were radiotracked from November 2009 to June 2011, resulting in 2,321 locations (591–937 locations per group) in 151 days of monitoring (69–81 days per group). The relationship between the number of locations and the cumulative home range presented a punctuated equilibrium, approaching multiple asymptotes with different sample size for each group (Fig. 1). This pattern may be explained by the shift of home-range areas during the wet season, associated with territorial expansion of the groups within the season.

Home range.—The linear river extent of home range of the 8 groups monitored during both dry seasons ranged from 1.8 to 22.9 km (Table 2). During the wet seasons, the linear home ranges for 5 groups varied from 14.8 to 31.7 km. On the basis of the 95% fixed-kernel method, during the dry season, the home-range size of 8 groups varied from 0.5 to 3.2 km^2 (Table 1), whereas the home-range sizes of the 5 groups monitored during the wet season varied from 1.0 to 12.0 km^2. However, these values may be underestimates due to differences in sampling effort and should be treated with caution. The 98% *k*-LoCoh home-range sizes (Fig. 2) for the radiotagged groups G2, G10, and G12 during the dry season were, respectively, 1.0, 2.3, and 0.1 km^2, whereas during the wet season they were 3.9, 7.9, and 3.6 km^2, respectively (Table 1), which represents an increase of 4 to 59 times in home-range size during the wet season.

The 3 radiotagged groups reared cubs during the monitoring period. Group G2 had 3 cubs during the wet season of 2010 (born in March), whereas the groups G10 and G12 had,

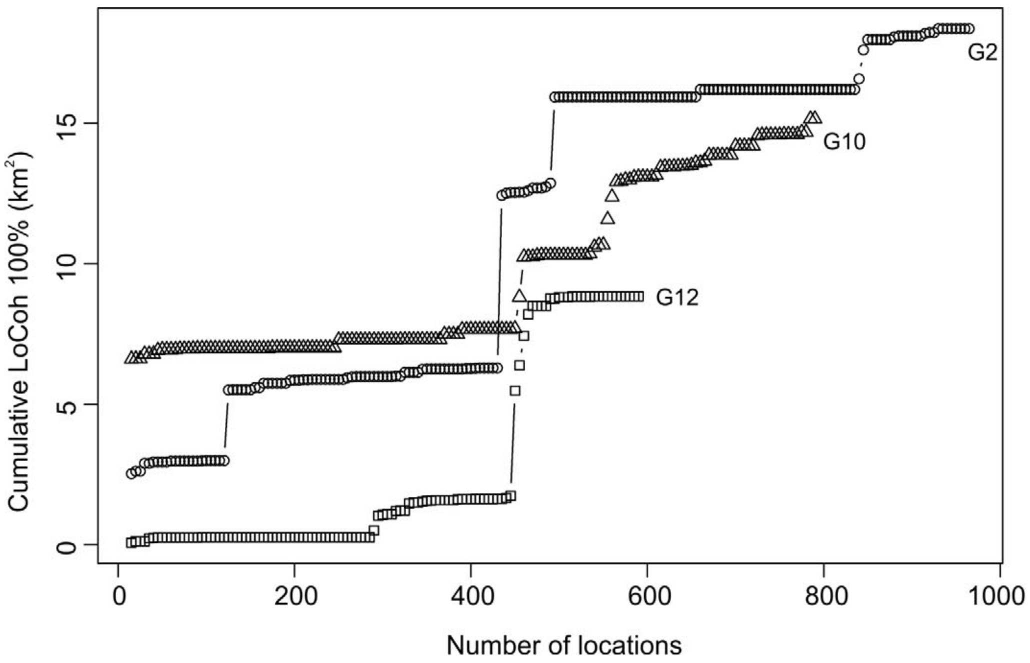

Fig. 1.—Cumulative estimated area used (LoCoh 100%) in relation to the number of chronological locations of 3 groups of giant otters radiotracked from November 2009 to June 2011, in the southern Pantanal of Brazil.

respectively, 6 and 2 cubs during the dry season of 2010 (born in September and August). Groups G10 and G12 had larger core areas during the wet season (*k*-LoCoh 50% = 0.5 and 0.01 km^2) than during the dry season, but group G2 reduced its core area by about 10 times from 0.03 to 0.003 km^2 between the dry season and the following wet season (Table 1). The daily speed of movement followed the same pattern, since during the wet season the mean daily speeds of groups G2, G10, and G12 were respectively 0.5 (0.03–1.9) km/h, 1.3 (0–4.7) km/h, and 0.8 (0.3–1.7) km/h, whereas during the dry season these values were 0.9 (0–3) km/h, 0.9 (0.04–4.6) km/h, and 0.4 (0.1–0.5) km/h.

Table 2.—Linear home-range estimate (km) of 10 giant otter groups monitored by radiotelemetry (RT) and direct observation (DO) during 4 seasons (DS 2009 = dry season of 2009, WS 2010 = wet season of 2010, DS 2010 = dry season of 2010, WS 2011 = wet season of 2011) from June 2009 to June 2011 in the southern Pantanal of Brazil.

Method	Groups	DS 2009	WS 2010	DS 2010	WS 2011
RT	G2	10.5	22.7		
	G10		9.1[a]	22.9	31.7
	G12			1.8	15.6
DO	G1	18.0	23.1	20.1[a]	6.4[a]
	G3	14.2	14.8	13.7	4.9[a]
	G4	17.2	6.7[a]	0.3[a]	4.1[a]
	G8	12.1			
	G9	13.0	8.5[a]		11.5[a]
	G11			9.4[a]	1.8[a]
	G13				1.6[a]
	Median	13.6	22.7	13.7	23.6

[a] Estimates should be accepted with caution, as they are based on few locations (<20), and therefore were not used to calculate the medians.

It was not feasible to monitor all groups during the wet season. Therefore, we estimated home-range fidelity only for the radiotagged groups and 2 other groups (G1 and G3). Home-range overlap varied from 0% to 87% between seasons (Table 1). The radiotagged groups G2 and G10 used 78% and 87%, respectively, of their dry-season home ranges (*k*-LoCoh 98%) during the consecutive wet season. The area occupied during the wet season of 2011 by group G12 did not overlap its home range in the previous dry season of 2010. Groups G2 and G12 both dispersed to flooded plains at the beginning of the wet season, abandoning the home ranges used during the previous dry season until the middle of the wet season. These groups used temporary streams and constructed dens and campsites on the banks of artificial ponds and on the roadside of the Estrada Parque Pantanal (EPP). The EPP is a dirt road with 1–2-m elevation that crosses a section of the southern Pantanal. From March to April 2011, the water level was at its highest and almost all riverbanks in the study area were submerged. During this time, groups G10 and G12 broke branches of emerged shrubs to construct clumsy nests, which the animals used to rest and defecate. The radiotagged groups were not neighbors during the study. Therefore, none of them overlapped the home range areas of other radiotagged groups in the same season. Groups G2 and G10 partially overlapped their own core areas (*k*-LoCoh 50%) in consecutive wet and dry seasons by 17% and 13%, respectively.

Selection of landscape features.—During the wet seasons, groups did not select any of the landscape categories to establish home ranges ($\Lambda = 0.05$, $P = 0.121$) or select landscape elements within the home ranges ($\Lambda = 0.059$, $P = 0.13$). During the dry season, selection for landscape features to establish home ranges differed significantly from random ($\Lambda =$

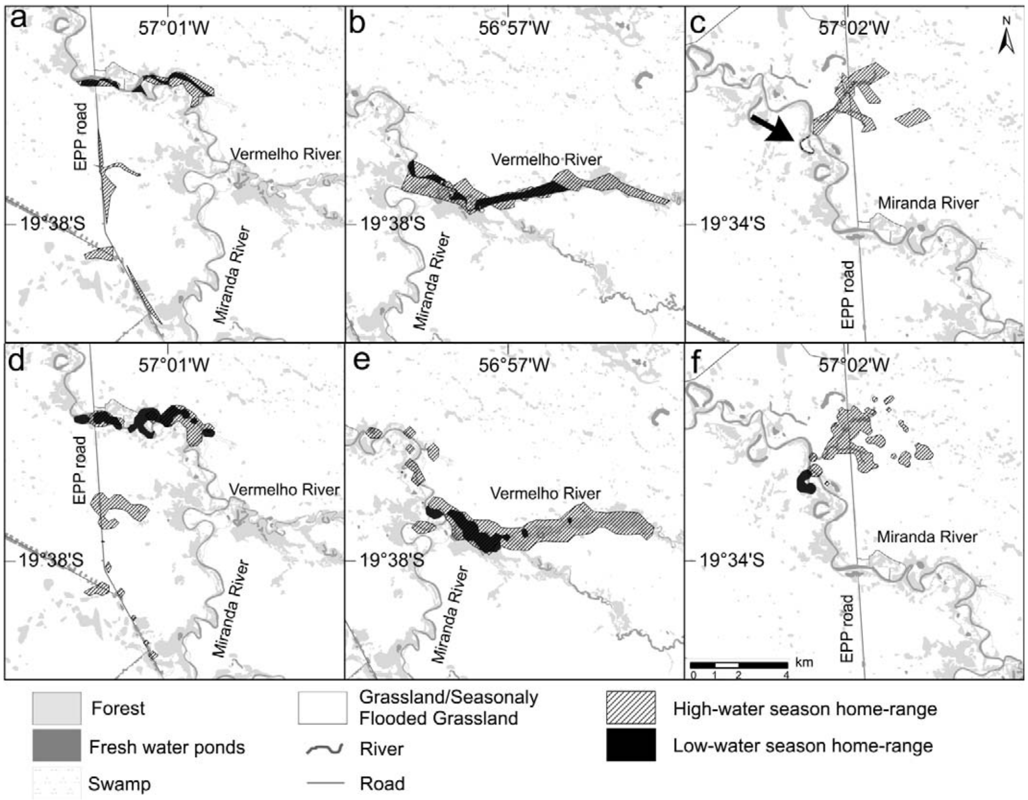

Fig. 2.—Seasonal home ranges of 3 giant otter groups monitored by radiotelemetry between November 2009 and June 2011, in the southern Brazilian Pantanal. Upper Figs.: *k*-LoCoh 98% of a) group G2, b) group G10, and c) group G12. The black arrow indicates the location of the reduced home range of group G12 during the dry season. Lower Figs.: Kernel 95% of d) group G2, e) group G10, f) group G12.

0.007, $P = 0.017$). The ranking matrix ordered the habitat types as river = forest = swamp = ponds > grassland (Table 3a). Changes in use of landscape features between seasons seemed to differ among groups. During the wet seasons, group G1 continued using the river more intensively than other landscape elements, whereas group G12 selected seasonally flooded grassland and grassland habitats. During the dry season, group G12 selected ponds, whereas the other groups selected the river and forest habitats (Fig. 3). Seasonally flooded grassland occurred within only 1 of the home ranges of the 8 groups studied during the dry seasons. Therefore, we excluded this landscape feature from the analyses related to this season.

Table 3.—Ranking matrix of habitat types (RI = river, PO = pond, FO = forest, SW = swamp, GL = grassland) selected by 8 giant otter groups during the dry seasons, from June 2009 to January 2011 in the southern Pantanal of Brazil. A) Proportional habitat use within group's kernel home ranges with proportion of total available habitat types within study area; B) proportions of independent locations for each group in each habitat type within group's kernel home range. Each mean element in the matrix was replaced by its sign; a triple sign represents significant deviation from random at $P < 0.05$. + indicates that the habitat was positively selected.

Habitat type	RI	FO	SW	PO	GL
A) Home range versus landscape					
RI	0	+	+	+	+++
FO		0	+	+	+++
SW			0	+	+++
FP				0	+++
GL					0
B) Radio locations versus home range					
RI	0	+	+++	+++	+++
PO		0	+	+	+++
FO			0	+	+++
SW				0	+
GL					0

During the dry season, giant otter groups did not select landscape elements within their home ranges ($\Lambda = 0.186$, $P = 0.07$), but the low probability for the null hypothesis indicates a likely type II error. The ranking matrix of landscape-element selection suggests that the river was proportionally more used than expected from availability relative to the other landscape elements (Table 3b). Forest was used mainly to build dens and campsites, and 83% of dens ($n = 156$) and 77 % of campsites ($n = 92$) were located in riparian forest (Table 4).

Discussion

Home range.—Despite increasing knowledge of the ecology of giant otters since the reference study by Duplaix (1980), data on the spatial ecology of the species has been restricted to observations made during the dry season, and most of these observations were reported as linear home ranges. Here we provide 2-dimensional as well as linear estimates of home

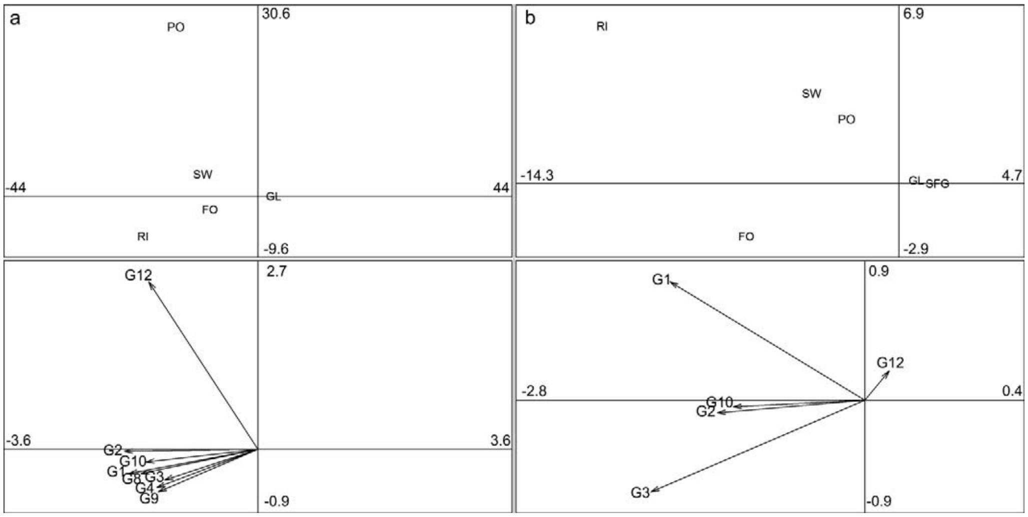

Fig. 3.—Results of eigenanalysis of landscape-element selection ratio by giant otter groups for 6 landscape elements (FO = forest, PO = ponds, GL = grassland, SFG = seasonal flooded grassland, SW = swamp, RI = river) from June 2009 to June 2011 in the southern Brazilian Pantanal. A) During the dry season (groups G1–G4, G8–G10, G12) and B) during the wet season (groups G1–G3, G10, G12). Upper graphs show the landscape-element loadings on the first 2 factorial axes and lower graphs show the groups' scores on the 1st factorial plane.

ranges for giant otter groups in an area of the southern Pantanal, in both dry and wet seasons. During the dry seasons, the linear home ranges varied from 1.8 to 22.9 km, with a median of 13.7 km and were of same magnitude as the linear home ranges for giant otters inhabiting areas in Guyana and the Amazon (Duplaix 1980; Evangelista and Rosas 2011a). Laidler (1984) suggested a home range of 32 km of creek or 20 km^2 of a lake in Guyana on the basis of the assumption that the groups cyclically move among different hunting places. However, such cyclic movements were not observed in our study site (Leuchtenberger and Mourão 2008) or elsewhere (Duplaix 1980; Evangelista and Rosas 2011a; Staib 2005). Using the 2-dimensional locations of our radiotagged groups, the home-range estimates for the dry season ranged from 0.1 to 2.3 km^2 (LoCoh 98%), which is similar to the 2-dimensional home ranges reported for giant otters in areas of the Amazon (0.6–1.1 km^2—Staib 2005; and 0.5–2.8 km^2—Utreras et al. 2005).

Home-range overlapping of radiotagged groups between seasons varied from 0% to 87%. During the wet season, 2 of the 3 radiotagged groups left the area they used during the dry season partially or entirely to move into the flooded plains. Seasonal shifts in home-range size have been observed for many carnivores (Curtis and Zaramody 1998; Dillon and Kelly 2008; Valenzuela and Ceballos 2000), including otters (Blundell et al. 2000), and seem to be strongly related to resource availability. Duplaix (1980) stated that giant otter groups abandon their ranges during the rainy season to follow dispersing fish into the flooded forest and swamps, and to search for higher banks for building dens and campsites. The availability of banks may not be restrictive, as the otters can use emerged shrubs to rest during flooding (this study). One group we radiotracked remained in its original stretch of river, but frequently used the flooded marginal areas. Giant otters can increase their home ranges at least 4-fold during the wet season in the Pantanal (this study) and in an area in the Amazon (Utreras et al. 2005) by taking advantage of the flooded areas along river courses.

Core areas comprised less than 7% of the home ranges of the groups and usually contained dens, latrines, and intensive foraging sites, as suggested by Duplaix (1980). During the 1st months of cub rearing, giant otter groups reduced their movements and limited their core areas to extremely small sizes (e.g., group G12 used a pond of 1.3 ha), as previously reported for the species (Duplaix 2004; Evangelista and Rosas 2011a; Laidler 1984) and other otters (Erlinge 1967; Hussain and Choudhury 1995; Melquist and Hornoker 1983; Ruiz-

Table 4.—Number of dens and campsites built by 10 giant otter groups in different landscape features (SW = swamp, GL = grassland, SFL = seasonal flooded grassland, FO = forest) during 4 seasons (DS 2009 = dry season of 2009, WS 2010 = wet season of 2010, DS 2010 = dry season of 2010, WS 2011 = wet season of 2011) from June 2009 to June 2011 in the southern Pantanal of Brazil.

	DS 2009		WS 2010		DS 2010		WS 2011		Total	
Habitat	Den	Campsite	Den	Campsite	Den	Campsite	Den	Campsite	Den	Campsite
SW	8	5	1	2	2	7	1	0	12	14
GL	5	2	1	2	2	0	2	2	10	6
SGL	0	0	2	1	0	0	2	0	4	1
FO	70	17	16	14	31	18	13	22	130	71
Total	83	24	20	19	35	25	18	24	156	92

Olmo et al. 2005). The restriction of movement and reduction of ranges during the first 4 months of cub rearing may be a strategy to improve the raising success, as this is the critical period for lactation and cub learning (Evangelista and Rosas 2011b), and cub mortality may be higher in this period (Schweizer 1992).

Selection of landscape features.—Changes in availability of landscape features may induce changes in habitat-selection patterns (Arthur et al. 1996; Humphrey and Zinn 1982). Giant otter groups were selective in relation to their use of landscape elements available during the dry season. However, they seemed to be less selective in positioning activity ranges during the wet season. According to Duplaix (1980), food availability is one of the key factors that affect habitat choice by giant otters. Therefore, when their prey becomes more dispersed through the floodplains, groups may move more unpredictably with regard to landscape elements when searching for food as a foraging strategy to maximize food gain, as is expected for an animal using an optimal foraging strategy (Schoener 1971).

During the wet season, giant otters were frequently observed fishing in the areas adjacent to the river, such as flooded forest, grassland, and swamps. These areas have shallow water, which is preferred for foraging by many otter species (Anoop and Hussain 2004; Hussain and Choudhury 1995; Kruuk 2006; Laidler 1984), presumably due to the higher concentration of prey during the flood season (Wantzen et al. 2002; Winemiller and Jepsen 1998). During the dry season, the river was most intensively used in relation to other landscape features that were available within the home range. However, there was variation in use of landscape elements between groups. Some groups selected marginal habitats, such as freshwater ponds and artificial ponds beside roads during the dry season, and seasonally flooded grassland during the wet season. However, this apparent preference may be an artifact of territoriality, since the groups that inhabited those marginal habitats were smaller than their neighboring groups. The use of such habitats by giant otter groups may be a result of lack of space in areas where the species has reached carrying capacity (Ribas et al. 2012). However, these marginal habitats may not support larger groups for long, as the fish stocks in these habitats are rapidly and drastically reduced due to the high rate of predation by piscivorous animals (Ribas et al. 2012) and deterioration of water conditions (Winemiller and Jepsen 1998).

The selection of forest by most of the groups during the dry season is probably related to the use of banks with vegetative cover near water bodies to build dens and campsites (Carter and Rosas 1997; de Souza 2004; Duplaix, 1980; Lima et al. 2012; Schenck 1999; Schweizer 1992). Banks covered by vegetation are less affected by erosion and may offer more protection of dens from predators (de Souza 2004; Lima et al. 2012). During the wet season, groups continued to use the highest forested banks available in their territories to build dens and campsites, and some groups used emerged shrubs to build temporary platforms in marginal flooded areas and swamps. The importance of vegetation cover has been noted for other otter species (*Lutrogale perspicillata*—Anoop and Hussain 2004; Nawab and Hussain 2012; *Lutra lutra*—Macdonald and Mason 1983; *Lontra provocax*—Medina-Vogel et al. 2003; *Hydrictis maculicollis, Aonyx capensis*—Perrin and Carugati 2000). The removal of riparian vegetation by canalization of rivers and streams has affected the habitat and prey of *L. provocax* in Chile and led to declines in density (Medina-Vogel et al. 2003). Vegetation cover on the bank seems to be important for all otter species (Anoop and Hussain 2004; Nawab and Hussain 2012) and should be considered as a key factor for the maintenance of giant otter groups.

Many studies have investigated the relationship between home-range size and individual or group mass and diet (e.g., Gittleman and Harvey 1982; Lindstedt et al. 1986; Ottaviani et al. 2006). On the basis of the relationship presented by Gittleman and Harvey (1982), social mustelids such as giant otters, sea otters (*Enhydra lutris*), and European badgers (*Meles meles*) have an unusually smaller home range than expected for a strict carnivore (Johnson et al. 2000). The home-range size of giant otter groups was similar to the home ranges estimates for other social otters as *Lutrogale perspicillata* (2.1 to 6.6 km^2—Hussain and Chudoury 1995), *H. maculicollis* (1.1 to 9.5 km^2—Perrin et al. 2000), and male groups of *E. lutris* outside the breeding season (0.6 to 1 km^2—Jameson 1989), despite differences in data analysis and on their social system. This suggests that the home ranges of giant otter groups in our study have a large and dense prey base that supports large otters in such small areas, which also demands healthy habitat. The maintenance of the annual hydrological fluctuations should be considered a priority for the conservation of vulnerable species that have fish as their principal prey, since the flood pulse is often linked to high fish productivity (Welcomme 1985, 1990). This is particularly a worry for giant otters in the Pantanal, because 70 small dams for hydroelectric purposes are planned and another 44 have already been constructed on streams that flow into the Paraguay River basin (Mourão et al. 2010); such alterations may cause drastic changes in the flood pulse of this large wetland.

Resumo

Ariranhas (*Pteronura brasiliensis)* vivem em grupos, que parecem abandonar seus territórios durante a estação de inundação. Nós estudamos a ecologia espacial de grupos de ariranhas durante as estações seca e chuvosa nos rios Vermelho e Miranda no Pantanal brasileiro. Nós monitoramos visualmente ou por rádio telemetria 10 grupos de ariranhas mensalmente entre junho de 2009 e junho de 2011. Nós estimamos o tamanho da área de vida de todos os grupos através dos seguintes métodos: 1) comprimento linear do rio, considerando as localizações extremas de cada grupo, e 2) kernel fixo. Para os grupos monitorados com telemetria nós também usamos o método 3) *k*-LoCoh. Fidelidade espacial e seleção de habitat dos grupos de ariranhas foi analisada sazonalmente. Baseado no método de k-LoCoh (98%), os tamanhos das áreas de vidas durante a estação chuvosa (3.6 –7.9 km^2) foram 4 a 59 vezes maiores do que durante as estações secas (0.1–2.3 km^2). Fidelidade de área de vida entre

estações variou de 0% to 87% entre os grupos de ariranhas e dois grupos monitorados com rádio telemetria dispersaram para áreas inundadas durante as estações chuvosas. Grupos de ariranhas foram seletivos em relação à composição da paisagem durante as estações secas, quando o rio foi mais intensamente utilizado em relação a outras características da paisagem. No entanto, eles pareceram ser menos seletivos no posicionamento de suas atividades durante as estações chuvosas. Durante essa estação, ariranhas foram freqüentemente observadas pescando em áreas adjacentes ao rio, como florestas inundadas, campos e brejos.

Acknowledgments

We thank CNPq (grant 476939/2008-9), the Rufford Small Grants Foundation (grant 88.08.09), the Mohamed bin Zayed Species Conservation Fund (project n. 10051040), and IDEA Wild for their financial support. We are also indebted to Embrapa Pantanal, Barranco Alto Farm, and the Federal University of Mato Grosso do Sul for their logistic support. Two of the authors (CL and LGROS) were recipients of CNPq scholarships. L. A. Pellegrin helped us with geographic information system processing. W. de L. e Silva, S. Benício, P. de Almeida, and J. A. D. da Silva assisted us in the field. L. Leuzinger, M. Schweizer, and J. Schweizer helped with aerial monitoring. We are particularly grateful to M. M. Furtado, M. A. F. Rego, and M. Bueno for their unstinting assistance and support during the capture and surgical procedures.

Literature Cited

Aebischer, N. J., P. A. Robertson, and R. E. Kenward. 1993. Compositional analysis of habitat use from animal radio-tracking data. Ecology 74:1313–1325.

Alho, C. J. R. 2008. Biodiversity of the Pantanal: response to seasonal flooding regime and to environmental degradation. Brazilian Journal of Biology 68(4, Suppl.):957–966.

Anoop, K. R., and S. A. Hussain. 2004. Factors affecting habitat selection by smooth-coated otters (*Lutrogale perspicillata*) in Kerala, India. Journal of Zoology (London) 263:417–423.

Arthur, S. M., B. F. J. Manly, L. L. McDonald, and G. W. Garner. 1996. Assessing habitat selection when availability changes. Ecology 77:215–227.

Blundell, G. M., R. T. Bowyer, M. Ben-David, T. A. Dean, and S. C. Jewett. 2000. Effects of food resources on spacing behavior of river otters: does forage abundance control home-range size? Pp. 325–333 in Biotelemetry 15: Proceedings of the 15th International Symposium on Biotelemetry (J. H. Eiler, D. J. Alcorn, and M. R. Neuman, eds.). International Society of Biotelemetry, Wageningen, Netherlands.

Blundell, G. M., J. A. K. Maier, and E. M. Debevec. 2001. Linear home ranges: effects of smoothing, sample size, and autocorrelation on kernel estimates. Ecological Monographs 71:469–489.

Callenge, C. 2006. The package "adehabitat" for the R software: a tool for the analysis of space and habitat use by animals. Ecological Modelling 197:516–519.

Callenge, C., and A. B. Dufour. 2006. Eigenanalysis of selection ratios from animal radio-tracking data. Ecology 87:2349–2355.

Câmara, G., R. M. C. Souza, U. M. Freitas, and J. Garrido. 1996. SPRING: integrating remote sensing and GIS by object-oriented data modelling. Journal of Computers & Graphics 20:395–403.

Carter, S., and F. C. W. Rosas. 1997. Biology and conservation of the giant otter *Pteronura brasiliensis*. Mammal Review 27:1–26.

Curtis, D. J., and A. Zaramody. 1998. Group size, home range use, and seasonal variation in the ecology of *Eulemur mongoz*. International Journal of Primatology 19:811–835.

De Solla, S. R., R. Bonduriansky, and R. J. Brooks. 1999. Eliminating autocorrelation reduces biological relevance of home range estimates. Journal of Animal Ecology 68:221–234.

De Souza, J. D. 2004. Estudos ecológicos da ariranha, *Pteronura brasiliensis* (Zimmermann, 1780) (Carnivora: Mustelidae) no Pantanal Mato-Grossense. Master's thesis, Federal University of Mato Grosso, Mato Grosso, Brazil.

Dillon, A., and M. J. Kelly. 2008. Ocelot home range, overlap and density: comparing radio telemetry with camera trapping. Journal of Zoology (London) 275:391–398.

Duplaix, N. 1980. Observations on the ecology and behavior of the giant otter *Pteronura brasiliensis* in Suriname. Revue Ecologique (Terre Vie) 34:495–620.

Duplaix, N. 2004. Guyana giant otter project: 2002–2004 research results. Oceanic Society. San Francisco, California, USA, 44 p. http://www.giantotterresearch.com/articles/OCEANIC_SOCIETY_Guyana_Project_Report_2002.

Erlinge, S. 1967. Home range of the otter *Lutra lutra* L. in southern Sweden. Oikos 18:186–209.

ESRI. 2010. ArcGIS—a complete integrated system. Environmental Systems Research Institute, Inc., Redlands, California. http://esri.com/arcgis. Accessed 10 August 2010.

Evangelista, E., and F. C. W. Rosas. 2011a. The home range and movements of giant otters (*Pteronura brasiliensis*) in the Xixuaú Reserve, Roraima, Brazil. IUCN Otter Specialist Group Bulletin 28:31–37.

Evangelista, E., and F. C. W. Rosas. 2011b. Breeding behavior of giant otter (*Pteronura brasiliensis*) in the Xixuaú Reserve, Roraima, Brazil. IUCN Otter Specialist Group Bulletin 28:5–10.

Ferreira, O., Jr. 2004. GPS track Maker. http://www.trackmaker.com. Accessed 1 January 2009.

Garcia de Leaniz, C., D. W. Forman, S. Davies, and A. Thomson. 2006. Non-intrusive monitoring of otters (*Lutra lutra*) using infrared technology. Journal of Zoology (London) 270:577–584.

Getz, W. M., and C. C. Wilmers. 2004. A local nearest-neighbor convex-hull construction of home ranges and utilization distributions. Ecography 27:489–505.

Gittleman, J. L., and P. H. Harvey. 1982. Carnivore home-range size, metabolic needs and ecology. Behavioral Ecology and Sociobiology 10:57–63.

Hamilton, S. K., S. J. Sippel, and J. M. Melack. 1996. Inundation patterns in the Pantanal wetland of South America determined from passive microwave remote sensing. Archiv für Hydrobiologie 137:1–23.

Hijmans, R. J., and J. van Etten. 2010. Raster: package for reading, writing, and manipulating raster (grid) type geographic (spatial) data. http://cran.r-project.org/web/packages/raster/index.html.

Humphrey, S. R., and T. L. Zinn. 1982. Seasonal habitat use by river otters and everglades mink in Florida. Journal of Wildlife Management 46:375–381.

Hussain, S. A., and B. C. Choudhury 1995. Seasonal movement, home range and habitat utilization by smooth-coated otter in National Chambal Sanctuary. Habitat 11:45–55.

Jameson, R. J. 1989. Movements, home range and territories of male sea ottersof Central California. Marine Mammal Science 5:159–172.

Johnson, D. D. P., D. W. Macdonald, and A. J. Dickman. 2000. An analysis and review of models of the sociobiology of the Mustelidae. Mammal Review 30:171–196.

Johnson, D. H. 1980. The comparison of usage and availability measurements for evaluating resource preference. Ecology 61:65–71.

Keitt, T. H., R. Bivand, E. Pebesma, and B. Rowlingson. 2010. Rgdal: bindings for the geospatial data abstraction library. R package version 0.6–24. http://CRAN.R-project.org/package=rgdal.

Kernohan, B. J., R. A. Gitzen, and J. J. Millspaugh. 2001. Analysis of animal space use and movements. Pp. 125–166 in Radiotracking and animal populations. (J. J. Millspaugh and J. M. Marzluff, eds.). Academic Press, San Diego, California.

Krebs, J. R., and N. B. Davies. 1997. Behavioural ecology: an evolutionary approach. 4th ed. Blackwell, Oxford, United Kingdom.

Kruuk, H. 2006. Otters: ecology, behavior and conservation. Oxford University Press Inc., New York.

Laidler, L. 1984. The behavioral ecology of the giant otter in Guyana. Master's thesis, University of Cambridge, Cambridge, United Kingdom.

Leuchtenberger, C., and G. Mourão. 2008. Social organization and territoriality of giant otters (Carnivora : Mustelidae) in a seasonally flooded savanna in Brazil. Sociobiology 52:257–270.

Leuchtenberger, C., and G. Mourão. 2009. Scent-marking of giant otter in the Southern Pantanal, Brazil. Ethology 115:210–216.

Lewin-Koh, N. J., et al. 2009. Maptools: tools for reading and handling spatial objects. R package version 0.7–29. http://CRAN.R-project.org/package=maptools.

Lima, D. dos S., M. Marmontel, and E. Bernard. 2012. Site and refuge use by giant river otters (*Pteronura brasiliensis*) in the Western Brazilian Amazonia. Journal of Natural History 46:729–739.

Lindstedt, S. L., B. J. Miller, and S. W. Buskirk. 1986. Home range, time, and body size in mammals. Ecology 67:413–418.

Macdonald, D. W. 1983. The ecology of carnivore social behaviour. Nature 301:379–384.

Macdonald, S. M., and C. F. Mason. 1983. Some factors influencing the distribution of otters (*Lutra lutra*). Mammal Review 13:1–10.

Medina-Vogel, G., V. S. Kaufman, R. Monsalve, and V. Gomez. 2003. The influence of riparian vegetation, woody debris, stream morphology and human activity on the use of rivers by southern river otters in *Lontra provocax* in Chile. Oryx 37:422–430.

Melquist, W. E., and M. G. Hornoker. 1983. Ecology of river otters in West Central Idaho. Wildlife Monographs 83:3–60.

Mourão, G., W. Tomas, and Z. Campos. 2010. How much can the number of jabiru stork (Ciconiidae) nests vary due to change of flood extension in a large Neotropical floodplain? Zoologia 27:751–756.

NASA Landsat Program, 2009. Landsat TM5, L5TM22607420090603, SLC-Off, INPE, São José dos Campos, 03 June 2009.

NASA Landsat Program, 2010. Landsat TM5, L5TM22607420100318, SLC-Off, INPE, São José dos Campos, 18 March 2010.

NASA Landsat Program, 2011. Landsat TM5, L5TM22607420110422, SLC-Off, INPE, São José dos Campos, 22 April 2011.

Nawab, A., and S. A. Hussain. 2012. Factors affecting the occurrence of smooth-coated otter in aquatic systems of the Upper Gangetic Plains, India. Aquatic Conservation Marine and Freshwater Ecosystems 22:616-625.

Ottaviani, D., S. C. Cairns, M. Oliverio, and L. Boitani. 2006. Body mass as a predictive variable of home-range size among Italian mammals and birds. Journal of Zoology (London) 269:317–330.

Perrin, M. R., I. D. Carranza, and I. J. Linn. 2000. Use of space by the spotted-necked otter in the KwaZulu-Natal Drakensberg, South Africa. South African Journal of Wildlife Research 30:15–21.

Perrin, M. R., and C. Carugati. 2000. Habitat use by the cape clawless otter and the spotted-necked otter in the KwaZulu-Natal Drakensberg, South Africa. South African Journal of Wildlife Research 30:103–113.

Powell, R. A. 2000. Animal home ranges and territories and home range estimators. Pp. 65–110 in Research techniques in animal ecology: controversies and consequences (L. Boitani and T. K. Fuller, eds.). Columbia University Press, New York.

R Development Core Team, 2011. R: a language and environment for statistical computing. R Foundation for Statistical Computing, Vienna, Austria, www.R-project.org. Accessed 01 July 2011.

Renard, D., and N. Bez. 2005. RGeoS: geostatistical package. R package. Version 2.1. Centre de Géostatistique, Ecole des Mines de Paris, Fontainebleau, France.

Reynolds, T. D., and J. W. Laundre. 1990. Time intervals for estimating pronghorn and coyote home ranges and daily movements. Journal of Wildlife Management 54:316–322.

Ribas, C. 2004. Desenvolvimento de um programa de monitoramento em longo prazo das ariranhas (*Pteronura brasiliensis*) no pantanal brasileiro. Master dissertation, Federal University of Mato Grosso do Sul, Brazil.

Ribas, C., G. Damasceno, W. Magnusson, C. Leuchtenberger, and G. Mourão. 2012. Giant otters feeding on caiman: evidence for an expanded trophic niche of recovering populations. Studies on Neotropical Fauna and Environment 47:19–23.

Rooney, S. M., A. Wolfe, and T. J. Hayden. 1998. Autocorrelated data in telemetry studies: time to independence and the problem of behavioural effects. Mammal Review 28:89–98.

Ruiz-Olmo, J., A. Margalida, and A. Batet. 2005. Use of small rich patches by Eurasian otter (*Lutra lutra* L.) females and cubs during the predispersal period. Journal of Zoology (London) 265:339–346.

Ryan, S. J., C. U. Knechtel, and W. M. Getz. 2006. Range and habitat selection of African buffalo in South Africa. Journal of Wildlife Management 70:764–776.

Samuel, M. D., D. J. Pierce, and E. O. Garton. 1985. Identifying areas of concentrated use within the home range. Journal of Animal Ecology 54:711–719.

Schenck, C. 1999. Lobo de Rio (*Pteronura brasiliensis*). Presencia, uso del hábitat y protección en el Perú. GTZ/INRENA, Lima, Peru.

Schoener, T. W. 1971. Theory of feeding strategies. Annual Review of Ecology and Systematics 2:369–404.

Schweizer, J. 1992. Ariranhas no pantanal: ecologia e comportamento de *Pteronura brasiliensis*. Edibran-Editora Brasil Natureza Ltda, Curitiba, Paraná.

Sikes, R. S., W. L. Gannon, and the Animal Care and Use Committee of the American Society of Mammalogists. 2011. Guidelines of the American Society of Mammalogists for the use of wild mammals in research. Journal of Mammalogy 92:235–253.

Silveira, L., et al. 2011. Tagging giant otters (*Pteronura brasiliensis*) (Carnivora, Mustelidae) for radio-telemetry studies. Aquatic Mammals 37:208–212.

Spencer, W. D. 2012. Home ranges and the value of spatial information. Journal of Mammalogy 93:929–947.

Stabler, B. 2003. Shapefiles, version 0.3. R package. http://cran.r-project.org.

Staib, E. 2005. Eco-etología del Lobo de Río (*Pteronura brasiliensis*) en el Sureste del Perú. PhD dissertation, Sociedad Zoológica de Francfort Perú. Lima, Peru.

Stamps, J. A. 1995. Motor learning and the value of familiar space. American Naturalist 146:41–58.

Thioulouse, J., D. Chessel, S. Doledec, and J. M. Olivier. 1997. Ade-4: a multivariate analysis and graphical display software. Statistics and Computing 7:75–83.

Tomas, W., P. A. L. Borges, H. J. F. Rocha, R. Sá Filho, F. Kutchenski, Jr., and T. V. Udry. 2000. Potencial dos Rios Aquidauana e Miranda, no Pantanal de Mato Grosso do Sul, para a conservação da ariranha (*Pteronura brasiliensis*). Pp. 1–12 in III Simpósio sobre Recursos Naturais e Sócio-econômicos do Pantanal: Os Desafios do Novo Milênio (Embrapa Pantanal, eds.), Corumbá, Brazil.

Utreras, V. B., E. R. Suárez, G. Zapata-Ríos, G. Lasso, and L. Pinos. 2005. Dry and rainy season estimations of giant otter, *Pteronura brasiliensis*, home range in the Yasuní National Park, Ecuador. Latin American Journal of Aquatic Mammals 4:1–4.

Valenzuela, D., and G. Ceballos. 2000. Habitat selection, home range, and activity of the white-nosed coati (*Nasua narica*) in a Mexican tropical dry forest. Journal of Mammalogy 81:810–819.

Wantzen, K. M., A. F. de Machado, M. Voss, H. Boriss, and W. J. Junk. 2002. Seasonal isotopic shifts in fish of the Pantanal wetland, Brazil. Aquatic Sciences 64:239–251.

Welcomme, R. L. 1985. River fisheries. FAO Fish. Technical Paper, 262.

Welcomme, R. L. 1990. Status of fisheries in South American rivers. Interciência 15:337–345.

Winemiller, K. O., and D. B. Jepsen. 1998. Effects of seasonality and fish movement on tropical river food webs. Journal of Fish Biology 53A:267–296.

Submitted 16 August 2012. Accepted 20 November 2012.

Associate Editor was Ricardo A. Ojeda.
